# Existence of various human parvovirus B19 genotypes in Chinese plasma pools: genotype 1, genotype 3, putative intergenotypic recombinant variants and new genotypes

**DOI:** 10.1186/s12985-016-0611-6

**Published:** 2016-09-17

**Authors:** Junting Jia, Yuyuan Ma, Xiong Zhao, Chaoji Huangfu, Yadi Zhong, Chi Fang, Rui Fan, Maomin Lv, Jingang Zhang

**Affiliations:** Laboratory for Viral Safety of National Centre of Biomedical Analysis, Beijing Institute of Transfusion Medicine, No. 27 Taiping road, Haidian District, Beijing, 100850 China

**Keywords:** Human parvovirus B19, Genotypes, Plasma pools, Recombination

## Abstract

**Background:**

Human parvovirus B19 (B19V) is a frequent contaminant of blood and plasma-derived medicinal products. Three distinct genotypes of B19V have been identified. The distribution of the three B19V genotypes has been investigated in various regions or countries. However, in China, data on the existence of different B19V genotypes are limited.

**Methods:**

One hundred and eighteen B19V-DNA positive source plasma pool samples collected from three Chinese blood products manufacturers were analyzed. The subgenomic NS1/VP1u region junction of B19V was amplified by nested PCR. These amplified products were then cloned and subsequently sequenced. For genotyping, their phylogenetic inferences were constructed based on the NS1/VP1-unique region. Then putative recombination events were analyzed and identified.

**Results:**

Phylogenetic analysis of 118 B19V sequences attributed 61.86 % to genotype 1a, 10.17 % to genotype 1b, and 17.80 % to genotype 3b. All the genotype 3b sequences obtained in this study grouped as a specific, closely related cluster with B19V strain D91.1. Four 1a/3b recombinants and 5 new atypical B19V variants with no recombination events were identified.

**Conclusions:**

There were at least 3 subtypes (1a, 1b and 3b) of B19V circulating in China. Furthermore, putative B19V 1a/3b recombinants and unclassified strains were identified as well. Such recombinant and unclassified strains may contribute to the genetic diversity of B19V and consequently complicate the B19V infection diagnosis and NAT screening. Further studies will be required to elucidate the biological significance of the recombinant and unclassified strains.

**Electronic supplementary material:**

The online version of this article (doi:10.1186/s12985-016-0611-6) contains supplementary material, which is available to authorized users.

## Background

Human parvovirus B19 (B19V), member of the Erythroparvovirus genus within the Parvoviridae family, is a widespread human pathogen that be associated with a broad range of clinical manifestations [1–4]. Infection of B19V in the normal population is usually asymptomatic or a self-limiting febrile illness, but can sometimes be life-threatening in high-risk populations, such as transient aplastic crisis (TAC) in patients with haematological disorders, chronic anemia in immunodeficient patients and abortion or hydrops fetalis in pregnant women.

B19V is a frequent contaminant of blood and plasma-derived medicinal products (PDMPs) [5–9]. Many reports documented the transmission of B19V infection by the administration of contaminated PDMPs, such as clotting factor concentrates, intravenous immunoglobulin (IVIG), intramuscular immunoglobulin (IMIG), and albumin, manufactured from a large number of plasma donations [10–13]. In order to ensure the quality and safety of PDMPs, since 2004, European Pharmacopoeia proposes NAT (nucleic acid testing) for B19V as an in-process test and stipulates a limit of 10^4^ IU/ml for manufacturing pools used for the manufacture of anti-D immunoglobulin and virus-inactivated pooled plasma [14]. U.S. FDA and the Plasma Protein Therapeutics Association (PPTA) recommended the same limit for levels of B19V in plasma pools destined for making all kinds of PDMPs in B19V NAT testing [15, 16]. One study demonstrated that, minipool NAT screening for B19V could effectively lower the prevalence and level of B19V in the final products and in the majority of cases render it undetectable and hence potentially reduce the risk of B19V transmission [17].

The genome of B19V consists of a single strand of linear DNA, about 5,600 nucleotides, which encodes a single nonstructural protein (NS1), multiple functional protein essential to viral replication and regulation of gene expression that is cytotoxic to host cells, and two structural proteins viral protein 1 (VP1) and viral protein 2 (VP2) [18, 19]. In addition, B19V also encodes two other smaller nonstructural proteins, 7.5 kDa and 11 kDa. The 11 kDa protein has been shown to have a role in virion production and trafficking in infected cells and also contributes to erythroid progenitor cell death during B19V infection, whereas the 7.5 kDa protein has not yet been reported to have functions during B19V infection [20, 21].

B19V is now formally subdivided into three distinct genotypes (1, 2, 3), which were defined as having approximately 10 % divergence in overall DNA sequence [22]. Furthermore, phylogenetic analyses have revealed two subgroups within genotypes 1 and 3 [23, 24]. Genotype 1 is represented by the prototype strain Au (GenBank Accession Number M13178) and Vn147 (GenBank Accession Number DQ357064), as the prototype strain for genotype 1a and 1b respectively. A recent study showed that two groups of genotype 1a co-existed globally, and that they were different in genome-wide synonymous positions. Thus it was proposed that the two groups of genotype 1a should be called subtype 1a1 and 1a2, respectively [25]. Genotype 2 is represented by the prototype strain A6 (GenBank Accession Number AY064475) and Lali (GenBank Accession Number AY044266). Genotype 3 is represented by V9 (GenBank Accession Number AX003421) and D91.1 (GenBank Accession Numbers AY083234), as the prototype strain for genotype 3a and 3b, respectively. All B19V genotypes appear to circulate but their relative frequency is strikingly different and their spatial and temporal distribution is not uniform [26]. Despite the variations among the genomes, these three B19V genotypes are assumed to have similar biological properties, pathogenic capacities, and transmission routes and pose a similar diagnostic challenge in the clinical setting [27, 28]. Nevertheless, recent reports presented evidence that genotype 2-specific sequences predominantly associated with heart tissue disease proposing that pathogenic properties might differ according to the genotype [29, 30]. Since all genotype variants of B19V can be contaminants of blood and PDMPs, the NAT assay as an in-process test used in the manufacturing of PDMPs must be able to detect all genotype variants. As genetic variation was to be expected in the future, the importance of depositing DNA sequence for B19V strains in the databases was emphasized in order to ensure that as much information is available, a good assay design as be enabled [31].

We previously did research on the prevalence of B19V in Chinese plasma pools [32]. Here, we identified the B19V genotypes in B19V-DNA positive plasma pool samples and reported the diversity of these B19V sequences.

## Methods

### Samples

A total of 118 B19V-DNA positive source plasma pool samples from three Chinese blood products manufacturers (A, B, and C) were included in the present study. These samples were confirmed positive for B19V DNA by an in-house qPCR assay adapted for all three genotypes of B19V [32]. Manufacturer A, B and C are located in central China, northern China and southwestern China, respectively. The plasma samples were collected between 2008 and 2013 (Table 1). Each batch of the plasma pools from the three manufacturers consisted of 2000 to over 8300 donations. Each donation mixed in the plasma pools was tested negative for anti-human immunodeficiency virus type 1/2 antibody (anti-HIV 1/2), hepatitis B surface antigen (HBsAg) and anti-hepatitis C virus antibody (anti-HCV) by ELISA, before pooling.

**Table 1:** Origin and characteristics of the 118 human parvovirus B19 sequences studied

Manufacture (Location in China)	No. of sequences	Yr(s) of sample collection	No. of samples with genotypes:					Mean genetic distance (%)^a^
			1a	1b	3b	intergenotypic recombinant ones	putative new genotypes	
A (Central China)	89	2008–2013	60	6	13	5	5	3.79
B (Northern China)	8	2008	0	0	7	1	0	0.45
C(South-western China)	21	2008	13	6	1	1	0	2.78
Total	118	2008–2013	73	12	21	7^b^	5	4.27

^a^Distance between all sequences from the same location

^b^Four of these recombinants were identified to be natural, while the others were supposed to be amplification artefacts

### DNA extraction and nested-PCR

DNA extraction from the source plasma pool samples was performed with the High Pure Viral Nucleic Acid Kit (Roche Diagnostics, Mannheim, Germany) according to the manufacturer’s instructions. Viral DNA for sequencing was prepared by nested-PCR amplification of a 1,100-bp region spanning the NS1-VP1u junction with the primers previously described by Servant et al. [22]. The PCR products were analyzed by electrophoresis in 1 % agarose gels and subsequently purified by using a TIANgel Midi Purification Kit (Tiangen Biotech, Beijing, China). Purified amplicons were cloned into the pMD18-T vector (TaKaRa Bio, Dalian, China) and a single clone derived from the PCR product of each sample was sequenced. As for samples suspected of recombination, over 20 additional clones derived from each sample were cloned and sequenced.

### Sequencing and phylogenetic analyses

The NS1/VP1-unique region of B19V positive samples were sequenced and included in the following phylogenetic analyses. Sequencing was carried out by the dideoxynucleotide chain termination method on an ABI 3730XL DNA Analyzer with BigDye® Terminator v3.1 Cycle Sequencing Kit in accordance with the manufacturer’s instructions (Applied Biosystems, Foster City, USA). Multiple sequence alignment was carried out through Clustal W. The reliability of the alignment was additionally checked by using the BioEdit program (Department of Microbiology, North Carolina State University, Raleigh, NC, USA; http://www.mbio.ncsu.edu/BioEdit/bioedit.html). The B19V genotypes were determined by phylogenetic tree analysis using MEGA version 6.0 and the 1069 nt NS1/VP1-unique region junction (positions 1884 to 2952 in M13178) proposed for genotyping [22]. One hundred and ninety-four non-redundant sequences spanning this region were downloaded from GenBank (December 2015) and included in the analysis. Genetic distances were calculated using the Kimura two-parameter method and phylogenetic trees were constructed by the neighbour-joining method. The reliability of clusters was evaluated using interior branch test with 1000 replicates. The sequences that were strongly positioned inside one of the genotype reference groups were compiled and their respective genotypes were assigned.

### Recombination analysis

The sequences suggestive of recombination by the above phylogenetic inferences that presented unclear clustering were subjected to recombination analysis, as described previously [33]. For this analysis, the bootscanning method implemented in SimPlot v.3.5.1 for Windows was used with the following parameters: window size 250 bp, step size 20 bp, F84 model of evolution (maximum likelihood) as the model to estimate nucleotide substitution, transition/transversion ratio of 2.0, and a bootstrap of 100 trees [33–35]. The significance threshold for the bootscan was set at 70 %. The positions of crossover sites were defined on the basis of the distribution of informative sites supporting the two incongruent topologies that maximized the chi-square value, a method implemented in SimPlot. Trees were displayed using MEGA version 6.0 software [36].

### Nucleotide sequence accession numbers

The GenBank accession numbers for the NS1-VP1u region of B19V reported in this study are KR819768-KR819885.

## Results

### Sequencing and phylogenetic analysis of sequences

**Fig. 1:**
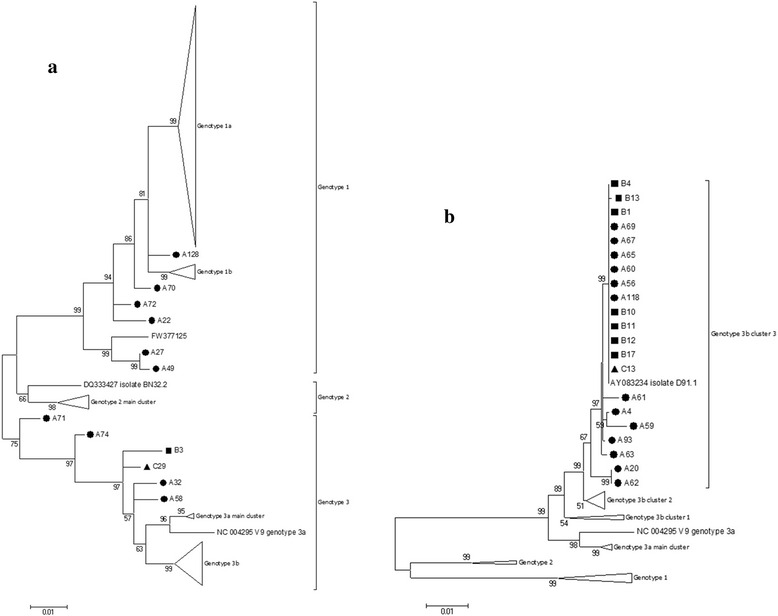
**a** Phylogenetic tree based on 312 sequences (118 obtained in this study and 194 retrieved from GenBank). **b** Phylogenetic tree based on 21 B19V-genotype 3 sequences from this study and a set of sequences downloaded from GenBank used as references for the different genotypes. Phylogenetic analyses are based on a region of 1069 nt and the neighbor-joining algorithm using the Kimura two-parameter model. The reference sequences are shown without symbols and are labeled with their GenBank accession number and isolate name. Sequences are from three Chinese blood products manufacturers: Manufacture A (*black circles*); Manufacture B (*black squares*); and Manufacture C (*black triangles*). Bootstrap replication frequencies ≥50 % are indicated above nodes. The basis of each isosceles triangle is proportional to the number of sequences. Branch lengths are drawn to scale

Sequencing and subsequent phylogenetic analysis were performed on a 1069-bp fragment (positions 1884 to 2952 in M13178) spanning part of the NS1 and VP1 gene of the B19V genome to determine the B19V genotype (Fig. 1). One hundred and eighteen sequences were obtained in this study, and designated according to its origin, for instance, A16 represented the B19V sequence amplified from the 16th sample from manufacturer A. Phylogenetic analysis of 118 sequences obtained in this study, together with 194 sequences retrieved from GenBank (December 2015), revealed the presence of B19V genotype 1 [91 of 118 (77.12 %)] and genotype 3 [27 of 118 (22.88 %)]. None of the samples clustered with the reference genotype 2 sequences (Fig. 1a).

As indicated in the phylogenetic tree, altogether 255 sequences belonging to genotype 1, including the 91 new sequences described here, with the mean genetic distance of 1.21 % and a maximal genetic distance of 5.60 % (KC013338 and A49, FW377125). Among the genotype 1 sequences, seventy-three new sequences together with 161 reference sequences clustered together to form the genotype 1a group, with the mean genetic distance of 0.89 % and a maximal genetic distance of 2.79 % (A45 and DQ293995, KC013321). Twelve new sequences together with two reference sequences clustered together to form the genotype 1b group, with the mean genetic distance of 1.11 % and a maximal genetic distance of 2.97 % (C33 and DQ357064). One new sequence (A128) displayed between the monophyletic clusters of genotype 1a and genotype 1b, suggesting the recombination between genotype 1a and 1b. Additionally, five new sequences (A70, A72, A22, A27 and A49) and one reference sequence (FW377125) from GenBank seemed to represent the outliers of genotype 1 (Fig. 1a).

Not a single genotype 2 virus was found in this study. Fourteen reference sequences from the GenBank formed the genotype 2 group, with the mean genetic distance of 1.66 % and a maximal genetic distance of 4.47 % (DQ333427 and AY064476).

The 53 sequences belonging to genotype 3, including the 27 new sequences described here, with the mean genetic distance of 1.94 % and a maximal genetic distance of 7.88 % (A71 and NC_004295). Six new sequences (A71, A74, B3, C29, A32 and A58) failed to group within the clusters of genotype 3a or 3b; they were suspected outliers or recombinants and were discussed later (Fig. 1a). With the exception of the outliers, all sequences fell into two groups (designated as genotype 3a and 3b, respectively). The mean intra-group distances were 0.95 and 1.16 %, respectively. Within genotype 3b, three main clusters were observed and all 21 new-found sequences fell into the single cluster (cluster 3). Moreover, seven of our 8 sequences from manufacture B belonged to this cluster (Fig. 1b).

### Recombination analysis of the unclassified sequences

A total of 12 new sequences could not be clearly assigned to a subgenotype by phylogenetic analysis described above. These sequences displayed distinct from the reference sequences from GenBank, implying they might be new subgroups or second-generation recombinants. These sequences were subjected to recombination analysis.

**Fig. 2:**
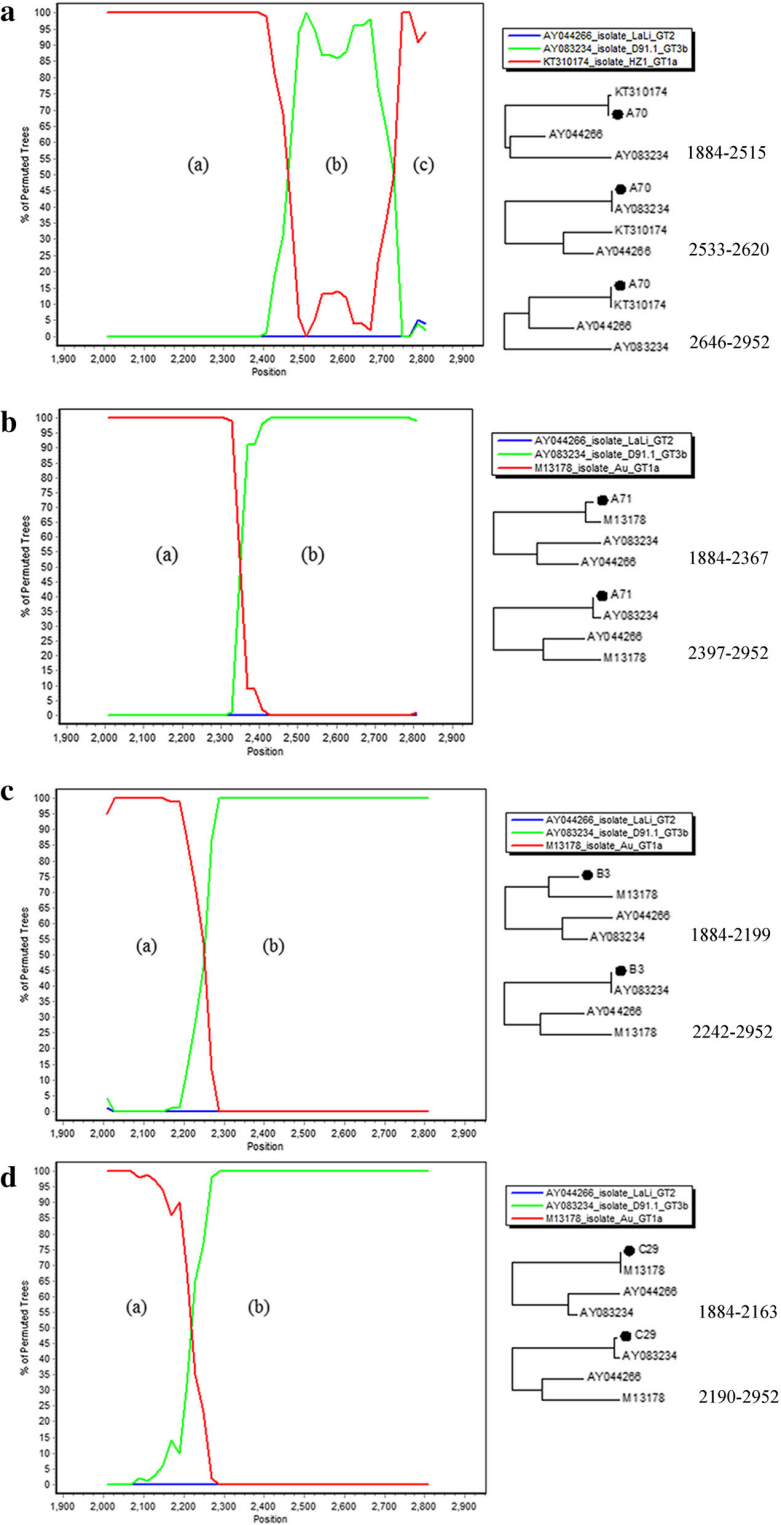
Analyses of four B19V 1a/3b recombinant sequences. Panel **a**, **b**, **c** and **d** represented the results of sequence A70, A71, B3 and C29, respectively. The left part of each panel was the results of bootscan analysis. At the right part of each panel were the neighbour-joining trees established on the basis of the fragments between breakpoints, as indicated by a bootscan plot of the sequence

Further analysis using SimPlot and bootscanning of the amplified fragment (1069-bp; positions 1884 to 2952 in M13178) indicated recombinant events between different genotypes. The breakpoints were further examined by constructing phylogenetic trees of the 2 nonrecombinant segments (Fig. 2 and Additional file 1: Figure S1).

Among the outliers of genotype 1, two sequences were identified to be the recombinants. A70 showed two recombinant events between genotype 1a (between nt 1884 and 2515), genotype 3b (between nt 2533 and 2620) and genotype 1a (between nt 2646 and 2952) (Fig. 2a). A72 showed two recombinant events between genotype 1a (between nt 1884 and 2531), genotype 3b (between nt 2600 and 2692) and genotype 1a (between nt 2744 and 2952) (Additional file 1: Figure S1a). In contrast, no recombinant signals were detected in the 4 remaining samples (A128, A22, A27 and A49) (exploratory tree analysis not shown), thus they were supposed to be putative new subtypes of genotype 1.

With regard to the outliers in genotype 3, three sequences were identified to be genotype 1a/3b recombinants. A71 showed a single recombinant event between genotype 1a (between nt 1884 and 2367) and genotype 3b (between nt 2397 and 2952) (Fig. 2b). B3 showed a single recombinant event between genotype 1a (between nt 1884 and 2199) and genotype 3b (between nt 2242 and 2952) (Fig. 2c). C29 showed a single recombinant event between genotype 1a (between nt 1884 and 2163) and genotype 3b (between nt 2190 and 2952) (Fig. 2d). Besides, the putative recombination signals were also detected in sequences A32 and A58 (Additional file 1: Figure S1b–c). A32 showed a single recombinant event between genotype 1a (between nt 1884 and 2068) and genotype 3b (between nt 2169 and 2952) (Additional file 1: Figure S1b). A58 showed a single recombinant event between genotype 3b (between nt 1884 and 2638) and genotype 1a (between nt 2744 and 2952) (Additional file 1: Figure S1c). However, as the breakpoints of these 2 sequences located either on the 5′ or 3′ end of each sequence, further analysis of longer sequences will be required to be demonstrative. In contrast, sequence A74 did not show any putative recombination signals, thus it was supposed to be putative new subtype of genotype 3.

### Further identification of the putative recombinants

A single plasma pool sample representing a mixture of thousands of plasma samples might easily contain B19V from different individuals and possibly of different genotypes. Thus, it was possible that the apparent recombinants described were in fact amplification artefacts if B19V of multiple origins coexisted in a sample. In order to explore if these recombinants are natural or not, we sequenced and analyzed more than 20 additional clones derived from the samples suspected of recombination (A32, A58, A70, A71, A72, B3 and C29). The genotypes of all these additional clones were listed in Additional file 2: Table S1.

For sample A70 and A71, all the additional clones derived from these 2 samples were identified as subtype 1a, while for sample B3, all were identified as subtype 3b. Furthermore, these 3 samples presented clones of the same genotype with nearly identical sequences, differences being related either to true quasispecies situation or to small errors during amplification. Although coexistence of subtype 1a and 1b have been identified in sample C29, none of the additional clones derived from this sample belonged to subtype 3b. As no coexistence of subtype 1a and 3b was found in each of the 4 samples described above, the artificial recombination of subtype 1a and 3b can hardly happen in these samples. Thus, B19V 1a/3b recombinants derived from these 4 samples in our study were supposed to be the natural recombinants rather than the laboratory artefacts.

As for the samples A32, A58 and A72, coexistence of subtype 1a and 3b and additional 1a/3b recombinants were identified in the additional clones. Sample A32 was a mixture of dominant subtype 1a (15 clones, with percent identity ranging from 96.7 to 100 %) and a minority of subtype 3b (2 clones, with 99.0 % sequence identity) and 3 different additional clones corresponding to be apparent recombinants. Sample A58 was a mixture of dominant subtype 3b (12 clones, with percent identity ranging from 98.1 to 100 %) and a minority of subtype 1a (9 clones, with percent identity ranging from 98.7 to 100 %) and 1 additional clone corresponding to be apparent recombinant. Sample A72 was a mixture of dominant subtype 1a (16 clones, with percent identity ranging from 96.4 to 100 %) and a minority of subtype 3b (3 clones, with percent identity ranging from 99.3 to 99.7 %) and 3 different additional clones corresponding to be apparent recombinants. These 6 additional suspected recombinant sequences described above, were different from original recombinant sequences derived from the same sample. In order to explore the possibility of amplification artefact occurred in samples A32, A58 and A72, the similarity and identity among the nucleotide sequences of the genotype 1a and 3b fragment involved in the recombinant event as defined by bootscanning and the non-recombinant genotype 1a and 3b fragment from the same plasma sample was investigated respectively. Results showed that in sample A32, nucleotide sequences of the subtype 1a and 3b fragment of the original recombinant sequence A32 and additional recombinant sequence A32_c1 were identical to that of non-recombinant subtype 1a and 3b fragment from the same plasma sample. Thus, the sequences A32 and A32_c1 were likely to be artificial recombinants between B19V subtype 1a and 3b strains coexistence in the sample A32. There were same scenarios for sequences A58, A72, A72_c3 and A72_c5. On the contrary, sequences A32_c19, A32_c20 and A58_c1 were supposed to be the natural recombinants.

In conclusion, four sequences (A70, A71, B3, and C29) were more likely to be bona fide recombinants. The other 3 samples (A32, A58 and A72) were containing multiple B19V strains originating from different samples in the plasma pool, thus the recombinants derived from these 3 samples were likely to be amplification artefacts.

## Discussion

Accumulating evidences have confirmed that the prevalence of each genotype varies in relation to geographic origin, population and sample type. Genotype 1, especially subtype 1a, is the most prevalent B19V currently circulating in most parts of the world and is represented by the prototype strain Au (GenBank Accession Number M13178) [37]. Genotype 2 is a rarely occurring genotype and has been sporadically found circulating in several European countries, Brazil and the United States with lower frequency and is represented by the prototype strain A6 (GenBank Accession Number AY064475) and Lali (GenBank Accession Number AY044266) [38–42]. Genotype 3 seems to be endemic to Ghana, but has also been found in Brazil, France, North India and the United States [22, 43–46]. Genotype 3 is represented by V9 (GenBank Accession Number AX003421) and D91.1 (GenBank Accession Number AY083234), as the prototype strain for genotype 3a and 3b respectively.

There is paucity of data on B19V genotypes circulating in China. Published data were limited to Ke’s study, in which the fragments approximately 1200 bp from NS1-VP1u region of five B19V-DNA positive blood donation samples were sequenced and these sequences were identified to be B19V genotype 1a by phylogenetic analysis [47]. In this study, we genotyped the B19V sequences in 118 B19V-DNA positive plasma pool samples. Using phylogenetic analysis, we demonstrated that at least three B19V subtypes, 1a, 1b and 3b, were currently present in China. In agreement with previous studies, our results indicated significantly well-defined subtypes within genotypes 1 and 3 [23, 48, 49]. Although the primers and probe used in this study were capable of detecting B19V genotype 2, no genotype 2 was detected. Absence of genotype 2 in this study was as expected, since this genotype was found relatively infrequently throughout the world. Alternatively, it is possible that mixtures of genotype 2 and other genotypes were present in the same sample, however, the identification might be compromised if the genotype 2 viral variants were present at a lower load or genotype 2 strains escaped detection because just one clone of each sample was sequenced and analyzed in this study, as discussed later in the limitations. Additionally, it is also possible that this was a result of limited sampling in restricted area.

In this study, phylogenetic analysis indicated B19V genotype 1 was predominant, with 91 of 118 B19V sequences (77.12 %) belonging to genotype 1. These results were in accordance with data collected from Vietnam, India, and Korea, suggesting the predominance of genotype 1 in Asia, and further confirming the predominance of genotype 1 in most parts of the world [24, 45]. Our study also presented the first report of B19V genotype 3 circulating in Chinese plasma donations. We identified 27 B19V genotype 3 sequences. Interestingly, all these new sequences except six outliers were subtyped as 3b and fell into the single cluster (genotype 3b cluster 3), most similar to strain D91.1 (AY083234) isolated in France. Moreover, seven of our 8 sequences from manufacture B belonged to this cluster. This suggested B19V subtype 3b might be endemic in certain regions of China. These observations might also suggest geographic differences in the spread of different B19V genotypes in diverse parts of China. However, these indications are based on relatively small numbers of samples collected in restricted area. Therefore, the data obtained in this study does at least partly reflect the status of B19V spreading in China.

The mutation rates of B19V are high for a DNA virus and comparable to those of RNA viruses [50, 51]. In accordance with a previous report, within the 1069-nt region, genotype 3 is the most diverse in this study, with a mean intragenotype distance of 1.94 %, followed by genotypes 2 (1.66 %) and 1 (0.89 % and 1.11 % for 1a and 1b respectively) [37]. They indicated that genotype 3 had a longer evolutionary history than the other two genotypes and that genotype 1 may be of a more recent origin [50]. Interestingly, the results of phylogenetic analysis indicated that genotype 3b sequences from China grouped as a specific, closely related cluster with B19V strain D91.1. Moreover, the further alignment and analysis showed that the genotype 3b sequences in this cluster were closely related to each other, with a genetic distance less than 1.61 %. These observations might suggest that B19V genotype 3b were introduced recently into China.

Besides the well-defined genotypes, the present study also suggested the circulation of B19V recombinants and few unclassified strains in China. We identified 7 putative B19V genotype 1a/3b recombinant viruses initially and further analyses indicated that 4 of these recombinants were natural while the other 3 were likely to be amplification artefacts. Two of these 4 natural recombinants (A70 and A71) were collected from manufacturer A located in central China where at least two different viral genotypes co-circulate, in accordance with the hypothesis that recombination of intra- and inter-B19V genotypes occurs in regions where different viral genotypes co-circulate [33]. Finding of these recombinants in our study was not surprising, as the frequent recombination in the parvoviruses and the frequent co-infection of more than one B19V strains, in some case co-infection of two different genotypes, allowing for the possibility of recombination [52–54]. A previous study by da Costa and colleagues has described the occurrence of 1a/3b recombinant in a single host and the recombination site located in the NS1 gene of B19V [33]. In a very recent publication, Shen et al. has analyzed 100 available NS1 and VPs genes of B19V from GenBank and found the recombination events between B19V genotype 1 and 2 as well as between genotype 1 and 3, leading to the recombinant cluster genotype 2, from which the recombinant breakpoints were found in NS1 or VPs of B19V [55]. In our study, B19V genotype 1a/3b recombinant viruses were identified, in accordance with da Costa’s and Shen’s reports. However, we did not detect the existence of B19V genotype 2 or genotype 1/2 recombinant viruses. Altogether, these findings confirmed the existence of intergenotypic recombinants of B19V. Such recombination may contribute to the genetic diversity of B19V, while whether recombination poses a real threat remains to be known. With regards to the other four unclassified sequences, we assumed they were putative new B19V genotypes or subtypes. In order to confirm their genotypes and elucidate the biological significance of the recombinant and unclassified strains, full-length genomic clones should be constructed and analyzed.

There are several limitations to this study. Above all, in view of the mere presence of B19V DNA in blood donor samples, as low as 0.003 %, in this paper we used the plasma pools as the samples for B19V genotyping [56]. The plasma pools were consisted of 2000 to over 8300 donations, thus there might be several B19V strains present in each sample, complicating data interpretation. Here in our study, just one of these strains in each sample was cloned and genotyped, limiting probably the accuracy of the prevalence analysis of different B19V genotypes. Besides, it was possible that the recombinant sequences derived from the large plasma pool samples were in fact amplification artefacts if B19V of multiple origins coexisted in a sample. Secondly, the lack of full-length sequences of B19V detected in this study, especially the recombinant and unclassified strains, was a limitation. Finally, at present there are 29 blood product manufacturers in China and our investigation was restricted in three of these. Thus, the data obtained in this study does not reflect the national circulating status of different B19V genotypes in China. Despite these limitations, our study represents the first report of the cocirculation of B19V 1a, 1b and 3b in China and the data implied the existence of putative B19V 1/3 recombinant and new strains.

## Conclusions

In conclusion, the present data provides, for the first time, evidence that B19V subtypes 1a, 1b and 3b are currently circulating in Chinese plasma donors, with genotype 1a predominating, as anticipated. Furthermore, this is the first report of the putative B19V 1a/3b recombinant and unclassified strains in China as well. Such strains may contribute to the genetic diversity of B19V and complicate the B19V detection and consequently have significant implications for B19V infection diagnosis and treatment, and for B19V NAT screening as well. Further studies will be required to elucidate the biological significance of the recombinant and unclassified strains.

## Additional files

Additional file 1: Figure S1. Analyses of three B19V 1a/3b recombinant sequences. The left part of each panel was the results of bootscan analysis. At the right part of each panel were the neighbour-joining trees established on the basis of the fragments between breakpoints, as indicated by a bootscan plot of the sequence. (JPG 4401 kb) Additional file 2: Table S1. Genotype of the additional clones from samples suspected of containing recombinant B19V. (DOCX 16 kb)

## Abbreviations

anti-HCV: anti-hepatitis C virus antibody
anti-HIV 1/2: anti-human immunodeficiency virus type 1/2 antibody
B19V: Human parvovirus B19
FDA: Food and Drug Administration
HBsAg: Hepatitis B surface antigen
IMIG: Intramuscular immunoglobulin
IVIG: Intravenous immunoglobulin
NAT: Nucleic acid testing
NIBSC: National Institute for Biological Standards and Control
NS1: Nonstructural protein
PDMPs: Plasma-derived medicinal products
Ph. Eur.: European Pharmacopoeia
PPTA: Plasma Protein Therapeutics Association
qPCR: Quantitative polymerase chain reaction
TAC: Transient aplastic crisis
VP1: Viral protein 1
VP2: Viral protein 2
WHO: World Health Organization
