# Supplementary figures and images for: Existence of various human parvovirus B19 genotypes in Chinese plasma pools: genotype 1, genotype 3, putative intergenotypic recombinant variants and new genotypes
Source: Virol J. 2016 Sep 17;13:155. doi: 10.1186/s12985-016-0611-6 (PMC5027099; doi:10.1186/s12985-016-0611-6)

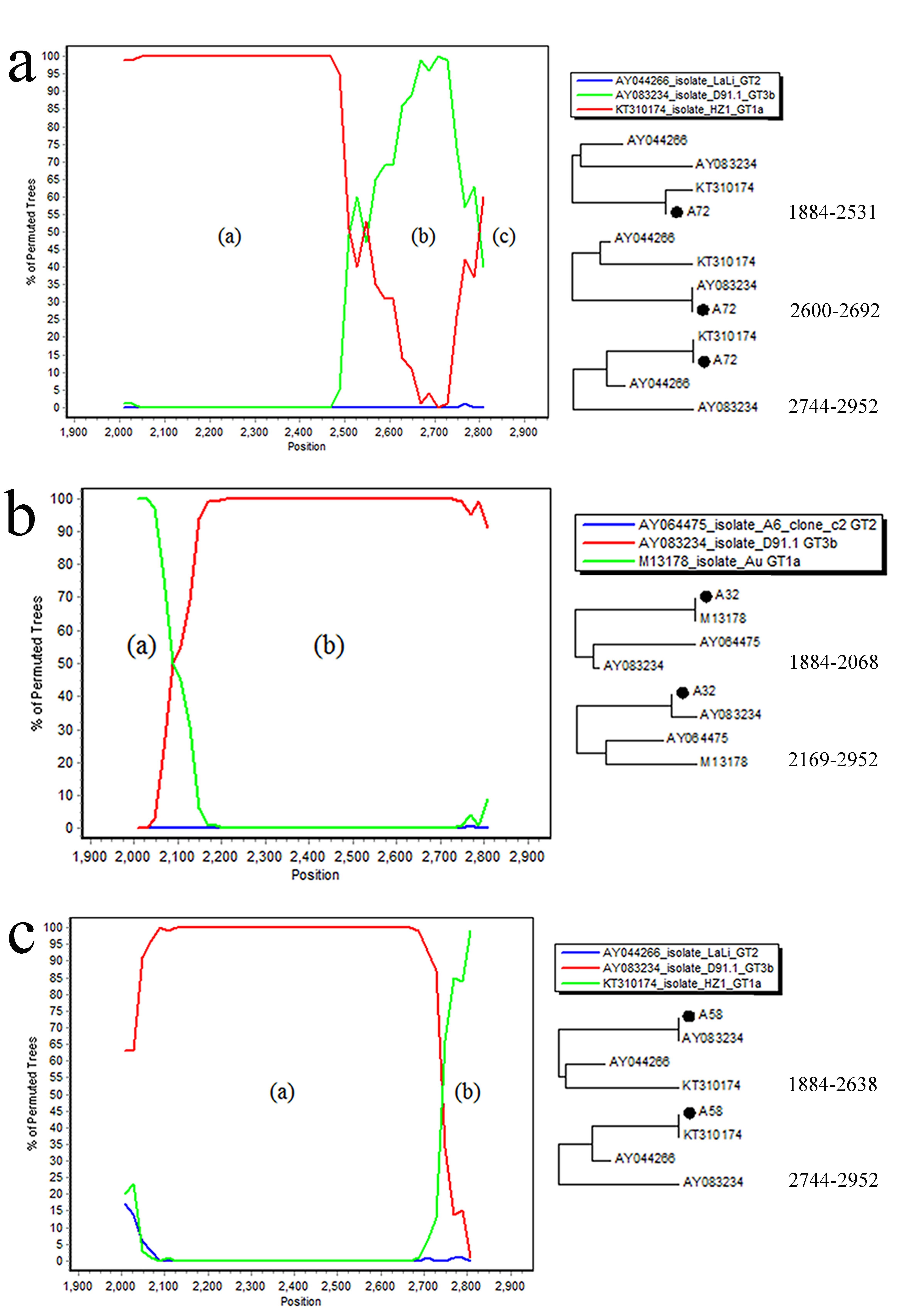

Supplement: Additional file 1: Figure S1. — Analyses of three B19V 1a/3b recombinant sequences. The left part of each panel was the results of bootscan analysis. At the right part of each panel were the neighbour-joining trees established on the basis of the fragments between breakpoints, as indicated by a bootscan plot of the sequence. (JPG 4401 kb) [file 12985_2016_611_MOESM1_ESM.jpg]
