# Supplementary material for: Existence of various human parvovirus B19 genotypes in Chinese plasma pools: genotype 1, genotype 3, putative intergenotypic recombinant variants and new genotypes
Source: Virol J. 2016 Sep 17;13:155. doi: 10.1186/s12985-016-0611-6 (PMC5027099; doi:10.1186/s12985-016-0611-6)
Supplement: Additional file 2: Table S1. — Genotype of the additional clones from samples suspected of containing recombinant B19V. (DOCX 16 kb) [file 12985_2016_611_MOESM2_ESM.docx]

**Table S1** Genotype of the additional clones from samples suspected of containing recombinant B19V

| **No. of the samples** | **No. of the additional clones** | **Genotype of the clones** |
| --- | --- | --- |
| A32 | 1 | 1a/3b recombinant |
|  | 2 | 3b (identical to a D91.1 strain isolated in France) |
|  | 3 | 1a |
|  | 4 | 1a |
|  | 5 | 1a |
|  | 6 | 1a (identical to clone 3 of A32) |
|  | 7 | 1a (identical to clone 3 of A32) |
|  | 8 | 1a (identical to clone 3 of A32) |
|  | 9 | 1a |
|  | 10 | 1a (identical to clone 3 of A32) |
|  | 11 | 1a |
|  | 12 | 1a (identical to clone 3 of A32) |
|  | 13 | 1a |
|  | 14 | 3b |
|  | 15 | 1a (identical to clone 3 of A32) |
|  | 16 | 1a (identical to clone 3 of A32) |
|  | 17 | 1a |
|  | 18 | 1a |
|  | 19 | 1a/3b recombinant |
|  | 20 | 1a/3b recombinant |
| A58 | 1 | 1a/3b recombinant |
|  | 2 | 3b |
|  | 3 | 3b (identical to a D91.1 strain isolated in France) |
|  | 4 | 3b (identical to a D91.1 strain isolated in France) |
|  | 5 | 3b |
|  | 6 | 3b |
|  | 7 | 1a |
|  | 8 | 3b |
|  | 9 | 1a |
|  | 10 | 3b |
|  | 11 | 1a (identical to clone 9 of A58) |
|  | 12 | 3b |
|  | 13 | 3b |
|  | 14 | 1a (identical to clone 9 of A58) |
|  | 15 | 1a (identical to clone 9 of A58) |
|  | 16 | 1a |
|  | 17 | 1a (identical to clone 9 of A58) |
|  | 18 | 3b |
|  | 19 | 3b |
|  | 20 | 1a (identical to clone 9 of A58) |
|  | 21 | 3b (identical to clone 12 of A58) |
|  | 22 | 1a (identical to clone 9 of A58) |
| A70 | 1 | 1a |
|  | 2 | 1a |
|  | 3 | 1a |
|  | 4 | 1a |
|  | 5 | 1a (identical to clone 4 of A70) |
|  | 6 | 1a (identical to clone 1 of A70) |
|  | 7 | 1a (identical to clone 1 of A70) |
|  | 8 | 1a (identical to clone 1 of A70) |
|  | 9 | 1a |
|  | 10 | 1a |
|  | 11 | 1a |
|  | 12 | 1a (identical to clone 11 of A70) |
|  | 13 | 1a (identical to clone 1 of A70) |
|  | 14 | 1a |
|  | 15 | 1a (identical to clone 1 of A70) |
|  | 16 | 1a |
|  | 17 | 1a (identical to clone 1 of A70) |
|  | 18 | 1a (identical to clone 1 of A70) |
|  | 19 | 1a (identical to clone 1 of A70) |
|  | 20 | 1a (identical to clone 1 of A70) |
|  | 21 | 1a (identical to clone 1 of A70) |
|  | 22 | 1a (identical to clone 1 of A70) |
| A71 | 1 | 1a |
|  | 2 | 1a |
|  | 3 | 1a |
|  | 4 | 1a |
|  | 5 | 1a (identical to clone 3 of A71) |
|  | 6 | 1a |
|  | 7 | 1a |
|  | 8 | 1a |
|  | 9 | 1a |
|  | 10 | 1a (identical to clone 3 of A71) |
|  | 11 | 1a (identical to clone 3 of A71) |
|  | 12 | 1a |
|  | 13 | 1a |
|  | 14 | 1a |
|  | 15 | 1a (identical to clone 3 of A71) |
|  | 16 | 1a (identical to clone 3 of A71) |
|  | 17 | 1a |
|  | 18 | 1a |
|  | 19 | 1a |
|  | 20 | 1a (identical to clone 4 of A71) |
| A72 | 1 | 1a |
|  | 2 | 1a |
|  | 3 | 1a/3b recombinant |
|  | 4 | 1a |
|  | 5 | 1a/3b recombinant |
|  | 6 | 1a (identical to clone 1 of A72) |
|  | 7 | 1a (identical to clone 1 of A72) |
|  | 8 | 1a (identical to clone 2 of A72) |
|  | 9 | 3b |
|  | 10 | 1a |
|  | 11 | 3b (identical to D91.1 strain isolated in France) |
|  | 12 | 3b |
|  | 13 | 1a |
|  | 14 | 1a |
|  | 15 | 1a (identical to clone 1 of A72) |
|  | 16 | 1a (identical to clone 1 of A72) |
|  | 17 | 1a |
|  | 18 | 1a (identical to clone 1 of A72) |
|  | 19 | 1 |
|  | 20 | 1a (identical to clone 1 of A72) |
|  | 21 | 1a |
| B3 | 1 | 3b (identical to a D91.1 strain isolated in France) |
|  | 2 | 3b (identical to a D91.1 strain isolated in France) |
|  | 3 | 3b (identical to a D91.1 strain isolated in France) |
|  | 4 | 3b (identical to a D91.1 strain isolated in France) |
|  | 5 | 3b (identical to a D91.1 strain isolated in France) |
|  | 6 | 3b (identical to a D91.1 strain isolated in France) |
|  | 7 | 3b (identical to a D91.1 strain isolated in France) |
|  | 8 | 3b (identical to a D91.1 strain isolated in France) |
|  | 9 | 3b (identical to a D91.1 strain isolated in France) |
|  | 10 | 3b (identical to a D91.1 strain isolated in France) |
|  | 11 | 3b (identical to a D91.1 strain isolated in France) |
|  | 12 | 3b |
|  | 13 | 3b (identical to a D91.1 strain isolated in France) |
|  | 14 | 3b |
|  | 15 | 3b (identical to a D91.1 strain isolated in France) |
|  | 16 | 3b (identical to a D91.1 strain isolated in France) |
|  | 17 | 3b (identical to a D91.1 strain isolated in France) |
|  | 18 | 3b (identical to a D91.1 strain isolated in France) |
|  | 19 | 3b (identical to a D91.1 strain isolated in France) |
|  | 20 | 3b (identical to a D91.1 strain isolated in France) |
|  | 21 | 3b (identical to a D91.1 strain isolated in France) |
| C29 | 1 | 1b |
|  | 2 | 1b (identical to clone 1 of C29) |
|  | 3 | 1a |
|  | 4 | 1a |
|  | 5 | 1b |
|  | 6 | 1b |
|  | 7 | 1b (identical to clone 1 of C29) |
|  | 8 | 1b (identical to clone 1 of C29) |
|  | 9 | 1b (identical to clone 1 of C29) |
|  | 10 | 1a (identical to clone 3 of C29) |
|  | 11 | 1a |
|  | 12 | 1b |
|  | 13 | 1b (identical to clone 1 of C29) |
|  | 14 | 1a |
|  | 15 | 1b (identical to clone 1 of C29) |
|  | 16 | 1b (identical to clone 1 of C29) |
|  | 17 | 1a (identical to clone 3 of C29) |
|  | 18 | 1b |
|  | 19 | 1a (identical to clone 3 of C29) |
|  | 20 | 1a |
|  | 21 | 1b |
|  | 22 | 1b (identical to clone 1 of C29) |
|  | 23 | 1b (identical to clone 1 of C29) |
|  | 24 | 1b (identical to clone 1 of C29) |
|  | 25 | 1b (identical to clone 1 of C29) |
|  | 26 | 1b |
